# Editorial: Biological nitrogen removal from low carbon wastewater

**DOI:** 10.3389/fmicb.2023.1137125

**Published:** 2023-02-28

**Authors:** Chongjun Chen, Rencun Jin, Wei Li, Lei Miao, Meng Zhang

**Affiliations:** ^1^School of Environmental Science and Engineering, Suzhou University of Science and Technology, Suzhou, China; ^2^Jiangsu Collaborative Innovation Center of Technology and Material of Water Treatment, Suzhou University of Science and Technology, Suzhou, China; ^3^Laboratory of Water Pollution Remediation, School of Engineering, Hangzhou Normal University, Hangzhou, China; ^4^School of Resources and Environmental Engineering, East China University of Science and Technology, Shanghai, China; ^5^School of Environmental Science and Engineering, Huazhong University of Science and Technology, Wuhan, China; ^6^College of Environmental and Resource Sciences, Zhejiang University, Hangzhou, China; ^7^Advanced Environmental Biotechnology Centre, Nanyang Environment & Water Research Institute, Nanyang Technological University, Singapore, Singapore

**Keywords:** biological nitrogen removal, low carbon wastewater, anammox, autotrophic Fe-driven process, wastewater treatment

In conventional nitrogen removal process, the deep nitrogen removal was realized by nitrification and denitrification for low carbon wastewater with external organic carbon addition, which would lead to higher cost, excessive sludge production and also greenhouse gas emission (Li et al., 2022). Carbon neutrality is an unprecedented challenge for the world, it is particularly necessary to invent innovative and economic process for nitrogen removal from low carbon wastewater (Bae and Kim, 2021; Rui and Peng, 2021). Recently, the relevant research is developing rapidly, including the carbon sources utilization, autotrophic nitrogen removal, novel microorganisms, and so on (Xie et al., 2022). For example, the Strass sewage plant in Austria (Nowak et al., 2015), the Changi sewage plant in Singapore (Trinh et al., 2021), and the new concept sewage plant in Yixing, China were all tried to achieve high-quality nitrogen removal from low carbon wastewater in the field.

The Frontiers Research Topic—Biological nitrogen removal from low carbon wastewater—invited contributions in the following areas: (a) low carbon wastewater treatment; (b) Biological nitrogen removal; (c) autotrophic nitrogen removal; (d) anaerobic ammonia oxidation (anammox); (e) innovative nitrogen removal biotechnology; (f) energy-carbon nexus analysis. In this Research Topic combined the total of 10 articles in this e-book to emphasis on the new findings and recent advances in various aspects of low carbon wastewater treatment.

In this topic, Jiang et al. systematically reviewed the potential ability of quorum sensing (QS) in the partial nitritation process using the control of free nitrous acid (FNA). Meanwhile, the QS would regulate the microbial community structure and function of sludge in sewage treatment systems. However, the metabolic network relationship, the molecular types of QS signals and the regulation of FNA on physiological functions of AOB and NOB should be needed for future study.

Kosgey et al. summarized the principles and advantages of several main nitrogen removal processes for low carbon wastewater, such as the partial nitrification/anammox (PN/A), autotrophic denitrification, nitritation-denitritation, and bioelectrochemical processes. In addition, the process kinetics, large-scale applicability and process configuration of the process were provided. However, the anammox-mediated processes were identified as the best alternative for nitrogen removal in low- or high-strength wastewaters treatment.

Pang et al. systematically summarizes abiotic and biotic nitrogen conversion process involving Fe, including nitrate/nitrite-dependent anaerobic Fe(II) oxidation (NDAFO) and anaerobic ammonium oxidation coupled with Fe(III) reduction (Feammox), and reviewed the biodiversity and microbial community structure of iron-oxidizing or -reducing microorganisms for nitrate/nitrite reduction or ammonium oxidation, respectively. In addition, the effects of pH, redox potential, Fe species, extracellular electron shuttles and natural organic matter were analyzed on the FeBNR reaction efficiency.

Wang et al. utilized the anoxic/aerobic/aerobic/anoxic (AOOA) process with fixed biofilms to improve nitrogen removal, and indicated the optimized running parameters including the volume ratio and dissolved oxygen (DO) concentration in the aerobic zone. In addition, the effluent could meet the discharge standard even in lower temperature and hydraulic retention time (HRT) for real domestic sewage treatment. This issue found that the main nitrogen removal pathway was elemental-sulfur-based autotrophic denitrification (ESAD), and proposed a new heoretical basis and research findings.

Wang et al. indicated the different filter (with different elemental sulfur powder, shell powder, and peanut hull powder) could achieve the differential operation effect for nitrate removal rate (NRR) using novel solid-phase carbon-sulfur-based composite filters. However, the research identified the HRT would considerably impact effluent quality after combined solid-phase-based mixotrophic denitrification process (SMDP) and anammox process. Meanwhile, the concentration of suspended solids (SS) of anammox effluent would also affect the performance of filters with the autotrophs and heterotrophs.

Feng et al. developed the zeolite membrane biological reactor (ZMBR) to enhance the PN process for iron oxide red wastewater (IORW) treatment. The results indicated the ZMBR could dramatically tolerate higher influent nitrogen loading rate (NLR) and nitrite production rate (NPR) than the traditional MBR. After the analysis found that the *Candidatus Kuenenia stuttgartiensis* was the main microorganism in the granular sludge, which would provide more proteins and lipids for better settle ability. The issue paper showed that zeolite addition remarkably enhanced the NLR and NPR, changed the composition and role of bacterial communities to increase capacity for nitrogen removal.

Jia et al. used a pilot-scale integrated fixed-film activated sludge (IFAS) reactor with one-stage partial nitritation/anammox (PN/A) process for real coal to ethylene glycol (CtEG) wastewater treatment, and better treatment effect was obtained. The authors found that the ammonia-oxidizing bacteria (AOB) and anammox bacteria (AnAOB) were co-action in flocs and biofilms respectively. Moreover, the issue paper found that the niche differentiation caused by morphological (DO) and spatial heterogeneity (gradient distribution of nutrients and toxins) maybe the main reason for the distribution of dominant microorganism. This issue paper provides support for enhancing microbial distribution and improving nitrogen removal efficiency.

Dong et al. indicated that the hydrogen pressure, pH, and biofilm thickness would dramatically influence the removal flux of ${\text{NO}}_{3}^{-}$-N, and obtained for the optimal control parameters. At the same time, the chemical and microbial mechanisms regulating the removal efficiency of MBfR surface biofilms were preliminarily identified through scanning and composition analysis of MBFR surface biofilms, combined with microbial community structure analysis. This issue paper provided a reference for the application and theoretical analysis of MBfR process.

Zhang M. et al. isolated a denitrifying bacterium named JM10B5aT (*Pseudomonas oligotrophica* sp. nov.) from the *juvenile Litopenaeus vannamei* aquaculture pond, and revealed that the sodium acetate was the optimum carbon source for denitrification process. In addition, the strain JM10B5aT could achieve complete nitrate removal with low C/N ratio. This issue paper indicated the main functional genes for JM10B5aT by genomic analyses, which provided the basis for the analysis of how the JM10B5aT participated in the complete denitrification process. However, how to promote the application of the strain in the actual wastewater engineering remained to be studied.

Zhang R. et al. indicated three ammonia nitrogen (AN) removal strains, designated as *Bacillusidriensis* CT-WN-B3, *Bacillus australimaris* CT-WL5-10, and *Pseudomonas oleovorans* CT-WL5-6 under alkaline conditions. At the same time, the AN removal efficiency of three strains under different pH, salinity and alkalinity was studied. The results of this paper were of great significance for understanding the physiological conditions of AN removal strains. However, the effects of multiple environmental factors need further study.

In general, this Research Topic not only reviewed the latest research progress of biological nitrogen removal from low carbon wastewater, but also developed a variety of suitable treatment processes in combination with laboratory tests and preliminarily explain of microbial mechanism. However, at present, there is a lack of pilot and engineering practice in biological nitrogen removal from low carbon wastewater. In the future, laboratory research and practical engineering application should be combined to promote engineering application with theoretical research.

## Author contributions

CC: supervision, funding acquisition, writing—review, and editing. RJ, WL, LM, and MZ: writing—review and editing. All authors contributed to the article and approved the submitted version.
